# Clinical and Objective Cognitive Measures for the Diagnosis of Cognitive Frailty Subtypes: A Comparative Study

**DOI:** 10.3389/fpsyg.2021.603974

**Published:** 2021-05-24

**Authors:** Qingwei Ruan, Weibin Zhang, Jian Ruan, Jie Chen, Zhuowei Yu

**Affiliations:** ^1^Shanghai Institute of Geriatrics and Gerontology, Shanghai Key Laboratory of Clinical Geriatrics, Huadong Hospital, Research Center of Aging and Medicine, Shanghai Institute of Geriatrics and Gerontology, Shanghai Medical College, Fudan University, Shanghai, China; ^2^Molecular and Cellular Biology Core Facility, Institute of Neuroscience, Chinese Academy of Sciences, Shanghai, China; ^3^Department of Geriatrics, Huadong Hospital, Shanghai Medical College, Fudan University, Shanghai, China

**Keywords:** neuropsychological test, mild cognitive impairment, pre*-*mild cognitive impairment subjective cognitive decline, physical pre*-*frailty, physical frailty, reversible cognitive frailty, potential reversible cognitive frailty

## Abstract

**Background:**

Cognitive frailty (CF) includes reversible and potentially reversible subtypes; the former is known as concurrent physical frailty (PF) and pre-mild cognitive impairment subjective cognitive decline (pre-MCI SCD), whereas the latter is known as concurrent PF and MCI. The diagnoses of pre-MCI SCD and MCI are based on clinical criteria and various subjective cognitive decline questionnaires. Heterogeneous assessment of cognitive impairment (CI) results in significant variability of CI, CF, and their subtype prevalence in various population-based studies.

**Objective:**

This study aimed to compare the classification differences in CI and CF subtypes from PF and normal cognition by applying clinical and objective cognitive criteria. Clinical criteria comprised Fried PF and clinical MCI criteria combined with the SCD questionnaire, whereas objective criteria comprised Fried PF and objective cognitive criteria based on the norm-adjusted six neuropsychological test scores.

**Methods:**

Of the 335 volunteers (age ≥ 60 years) in this study, 191 were diagnosed with CI based on clinical cognitive diagnosis criteria, and 144 were identified as robust normal based on objective cognitive assessment from the community-dwelling older adult cohort. Individuals with clinical CI, including 94 with MCI and 97 with pre-MCI SCD, were reclassified into different *z*-score-derived MCI, pre-MCI SCD, and normal subgroups based on objective cognitive criteria. The classification diagnostic accuracy of normal cognition, PF, pre-MCI, MCI, CF, and CF subtypes based on clinical and objective criteria was compared before and after adjusting for age, sex, and education level.

**Results:**

The reclassification of objective assessments indicated better performance than that of clinical assessments in terms of discerning CI severity among different subgroups before adjusting for demographic factors. After covariate adjustment, clinical assessments significantly improved the ability to cognitively discriminate normal individuals from those with pre-MCI SCD and MCI but not the *z*-score-derived pre-MCI SCD and MCI groups from the robust normal group. Furthermore, the adjustment did not improve the ability to discriminate among individuals with reversible CF from those with potentially reversible CF and pre-MCI only SCD from MCI only SCD.

**Conclusions:**

Objective criteria showed better performance than clinical criteria in the diagnosis of individuals with CI or CF subtypes. Rapid clinical cognitive screening in combination with normative *z*-scores criteria is cost effective and sustainable in clinical practice.

## Introduction

Cognitive frailty (CF) is defined as a clinical disorder with concurrent physical frailty (PF) and cognitive impairment (CI) but without dementia (Kelaiditi et al., 2013). Numerous epidemiological studies have shown that PF increases the risk of future cognitive decline and all-type dementia (Robertson et al., 2013; Kojima et al., 2016; Ma et al., 2019; Wallace et al., 2019). The combination of PF/physical prefrailty (PPF) and CI could better assess the risk for adverse outcomes in older adults (Forti et al., 2014; Yu et al., 2018; Aliberti et al., 2019; Meiner et al., 2020). CF can further be classified into two subtypes: reversible CF (RCF) and potentially reversible CF (PRCF). RCF is an optimal target for preventing elderly dependence (Ruan et al., 2015). However, the incidences of RCF and PRCF reportedly vary—from 2.5 and 1% to 19.86 and 6.3%, respectively—according to various population-based studies (Panza et al., 2018; Sugimoto et al., 2018; Ruan et al., 2020a). The use of different CF models, such as PPF or premild CI (MCI) subjective cognitive decline (SCD) that includes early (or cognitive compensation stage) and two later stages of subtle cognitive decline (Jessen et al., 2014), is considered a cause of such diversity; moreover, the different tools available for the assessment of PF have contributed to a marked variability in results (Canevelli and Cesari, 2017). The PF phenotype that includes PPF and PF, developed in the US Cardiovascular Health Study, is widely used by researchers to screen PF. Typically, CF models only contain the PF phenotype that was assessed using these modified criteria (Fried et al., 2001). Most studies involved PRCF resulting from the combination of PF and MCI. Only two studies have involved RCF resulting from the combination of PF and pre-MCI SCD (Solfrizzi et al., 2017) and that of PF/PPF and pre-MCI SCD (Ruan et al., 2020a).

Another more important cause of the significant variability of CF prevalence in various population-based studies is the heterogeneous assessment of CI. For the assessment of MCI, major studies have adopted global cognition screening measures, such as the Mini-Mental State Evaluation (MMSE) and the Montreal Cognitive Assessment (MoCA), to diagnose CI according to predetermined cutoff ranges (Trzepacz et al., 2015; Bosco et al., 2017, 2020). Although these measures can assess cognitive performance in different cognitive domains, the MMSE was less likely to detect early MCI because of ceiling effects in healthy controls (Trzepacz et al., 2015). The MoCA is a more difficult test than the MMSE, has fewer floor and ceiling effects, and is more sensitive than the latter for the detection of early cognitive decline (Larner, 2012; Trzepacz et al., 2015). However, the MoCA may yield scores lower than the cutoff values owing to the effect of demographic factors (Rossetti et al., 2011). A Clinical Dementia Rating (CDR) score of 0.5 is unlikely to differentiate MCI severity and MCI subtypes (Chang et al., 2011). To date, only two studies have adopted pre-MCI SCD for RCF diagnosis (Solfrizzi et al., 2017; Ruan et al., 2020a). Although the pre-MCI SCD criteria frame proposed by the SCD-I Working Group (Solfrizzi et al., 2017) was adopted, it is not suitable for the identification of slightly abnormal cognitive performance or subtle cognitive decline. Furthermore, pre-MCI SCD diagnosis in major studies is based on various self-reported questionnaires related to the memory domain (Rami et al., 2014; Rabin et al., 2015). Therefore, establishing objective cognitive criteria is essential for accurately diagnosing CI, CF, and their subtypes.

In the past few years, normative *z*-scores on the neuropsychological test battery have been established based on data collected in a cognitively normal population (Weintraub et al., 2009; Weintraub et al., 2018; Ruan et al., 2020b). It was possible to objectively identify pre-MCI SCD, MCI, and MCI subtypes. The false-positive diagnostic errors caused by the clinical MCI criteria (Edmonds et al., 2015a,b) significantly decreased using normative *z*-scores of domain-specific tests. When process *z*-scores obtained from learning and memory tests were integrated into non-invasive objective criteria, early pre-MCI SCD could be objectively diagnosed (Thomas et al., 2018). Moreover, patients with objective pre-MCI SCD showed early entorhinal pathological changes and faster amyloid accumulation but less widespread medial temporal neurodegeneration than the observations in patients with MCI (Thomas et al., 2020). Therefore, if objective criteria for MCI and pre-MCI SCD are integrated into the CF criteria, the accuracy of the diagnosis of CF subtypes would significantly improve.

The differentiating factor between the clinical and objective criteria is the number of tasks used—only screening tests versus additional tests for each cognitive domain. Objective criteria are more effective when they are based on additional cognitive tests. The present study aimed to explore the diagnostic accuracy of CI, CF, and their subtypes by comparing the discordance between clinical [clinical MCI criteria combined with the Spanish SCD questionnaire (SCD-Q) MyCog scores] (Rami et al., 2014) and objective assessments of CI in different subgroups based on cognitive status and CF stratifications.

## Materials and Methods

### Participants

Overall, 367 volunteers (age ≥ 60 years) were recruited from communities across Shanghai via face-to-face communication in each setting. Clinical assessment and neuropsychological tests were conducted from September 2018 to June 2019. After excluding individuals with severe disability, complete loss of hearing and vision, and dementia, 335 eligible individuals (age ≥ 60 years) were included in the study. Among these participants, 94 met the clinical criteria for MCI (Petersen et al., 2001), and 97 met the criteria for pre-MCI SCD based on the SCD-Q MyCog scores (Rami et al., 2014) after excluding individuals with MCI. Furthermore, 144 robust normal individuals having at least 1 year of follow-up data and meeting the normal cognition criteria based on objective diagnosis (the *z*-scores of six neuropsychological tests) at the second annual study visit were used as controls in the study (Sliwinski et al., 1996; Ruan et al., 2020b). The 191 participants diagnosed with MCI or pre-MCI SCD using clinical cognitive criteria were further reclassified by objective cognitive diagnosis. This study was approved by the Ethics Committee of Huadong hospital, and written informed consent was obtained from each volunteer or authorized representative.

### Clinical Evaluation

Participants were classified as MCI if they met the following criteria: (1) subjective memory complaint if their MyCog score was ≥7 (Rami et al., 2014); (2) CDR score of 0.5; (3) an MMSE score of 19–30 for education levels (cutoff scores: >19 for illiteracy, >22 for primary school, and >26 for middle school and above; Petersen et al., 2001); (4) no or minimal impairment in activities of daily living as determined by a clinical interview with the patient and informant (Lawton and Brody, 1969); and (5) not demented based on the *Diagnostic and Statistical Manual of Mental Disorders*, Fourth Edition (American Psychiatric Association, 1994). Participants were classified as pre-MCI SCD if their MyCog score was ≥7 (Rami et al., 2014) after excluding MCI. Depression symptomatology was excluded using the short form of the Geriatric Depression Scale (GDS) (Chau et al., 2006). The cognitive and non-cognitive subscales of the Alzheimer’s Disease Assessment Scale (ADAS-Cog and ADAS-Non-cog, respectively) were used to evaluate the severity of CI and behavior alteration (Rosen et al., 1984). The self-report severity scores based on a brief version of the Neuropsychiatric Inventory Questionnaire (NPI-Q) were used to evaluate the severity of neuropsychiatric symptoms (Kaufer et al., 2000).

### Neuropsychological Evaluation

According to the criteria reported in the literature with minor modification (Edmonds et al., 2015b; Thomas et al., 2018), MCI and pre-MCI SCD status were assessed using the normative *z*-scores of six neuropsychological tests and process *z*-scores of the Hopkins Verbal Learning Test-Revised (HVLT-R) (Ruan et al., 2020b). The tests are as follows: Trail Making Test A and B (TMT A and B) for executive or attention domain; Boston Naming Test and Animal List generation for language domain; HVLT-R for memory domain, including delayed free correct responses and HVLT-R recognition; and three process scores from the HVLT-R for identifying early pre-MCI SCD. Briefly, the HVLT-R is a 12-item (4 words from 3 semantic categories) word-list learning and memory test that includes three learning trials (List A, Trials 1–3); an interference trial with a different list (List B); a short-delay free recall trial (Trial 4) for List A; a long-delay free recall trial (Trial 5) for List A after 25 min; and delayed recognition of 24 words, including 12 List A words and 6 related and 6 unrelated non-List A words. The three process scores from the HVLT-R included the following: learning slope [(List A Trial 3–List A Trail 1)/3], retroactive interference (List A Trial 4/List A Trial 3), and intrusion errors (total number of extralist intrusion errors across all recall trials). Other neuropsychological test batteries, including digit span forward or backward and digit symbol (Ruan et al., 2020b), to assess attention/processing speed domain were also performed for verifying the correction of objective criteria based on six previous neuropsychological test batteries and three memory process scores.

### MCI and Pre-MCI SCD Evaluation by Normative *Z*-Scores

All raw total or process scores were converted to age-, education-, and sex-adjusted *z*-scores based on regression coefficients derived from our robust normal samples (Ruan et al., 2020b). If a participant had *z*-scores of >1 standard deviation (SD) from the norm on TMT A, TMT B, and intrusion errors or *z*-scores of <1 SD from the norm on the other measures of six neuropsychological test batteries, the individual was considered to have an impaired total score (the normative *z*-scores of six neuropsychological tests) or process score (Supplementary Table 1). The 191 participants with CI as diagnosed by clinical criteria were further classified by *z*-scores as pre-MCI SCD [two impaired process scores or one impaired process score and one impaired total score or impaired total score on two measures across different cognitive domains or Functional Assessment Questionnaire (FAQ) score of 6–8] or MCI [impaired total score on two measures in the same domain or one impaired score in each of three cognitive domains (memory, executive function, and language domains) or FAQ score of ≥9]; the remainder were classified as cognitively normal after exclusion of CI.

### PF Evaluation

The five-item Fried PF scale (fatigue, weakness, slowness, low physical activity, and weight loss) with Chinese reference values (Hao et al., 2017) was used to assess PF phenotypes in the sample. Scores on the Fried PF scale ranged from 0 to 5, with scores of 1–2 representing pre-frail and 3–5 representing frail.

### Evaluation of CF Subtypes

Participants were classified as RCF if they had both PPF/PF and pre-MCI SCD and as PRCF if they had both PPF/PF and MCI (Ruan et al., 2015).

### ApoE Genotyping

Two single-nucleotide polymorphisms (rs429358 and rs7412) were genotyped to identify the APOE genotypes containing the APOE ε2, ε3, and ε4 alleles using a SNaPshot minisequencing assay from peripheral whole blood samples (Kim et al., 2010). The individuals were divided into the following subgroups according to the frequency of ε4: 0, 1, and 2.

### Statistical Analysis

All continuous variables were assessed using one-sample Kolmogorov–Smirnov test and were deemed to be non-normally distributed. Descriptive statistics were reported as medians and interquartile ranges for continuous variables and as absolute numbers and percentages for categorical variables. The differences among the demographic, neuropsychological, and clinical characteristics of subgroups based on objective cognitive criteria were compared using the Kruskal–Wallis and chi-squared tests. Statistical significance was determined using a cutoff *P*-value of 0.050. These multiple comparisons of clinical and objective measures were further analyzed using nominal regression analyses after adjusting for age, sex, and education level. Data were analyzed using the SPSS 18.0 software.

## Results

### Comparison Between Clinical and Objective Assessments of CI

**FIGURE 1:**
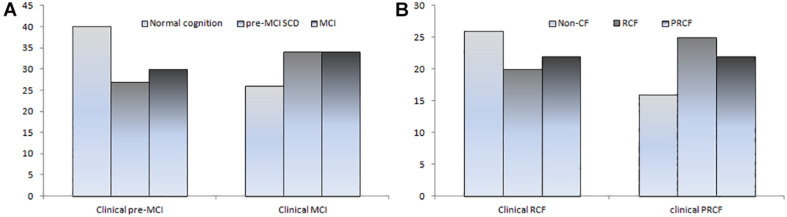
Number of CI **(A)** and CF subtypes **(B)** based on clinical criteria was reclassified according to objective cognitive criteria.

The 191 participants with pre-MCI SCD or MCI diagnosed using clinical cognitive criteria were divided into *z*-score-derived pre-MCI SCD and MCI and *z*-score-derived normal subgroups based on objective cognitive criteria. The characteristics of the three subgroups and robust normal controls are presented in Table 1. Age in the *z*-score-derived MCI group was higher than that in the *z*-score-derived normal groups (*P* < 0.05). The education level of the *z*-score-derived MCI and pre-MCI SCD groups was significantly lower than that of the robust normal control group (*P* < 0.001); furthermore, the education level of the *z*-score-derived MCI group was lower than that of the *z*-score-derived normal groups (*P* < 0.01). The frequency of ApoE ε4 was significantly lower in the robust normal control group, whereas ApoE ε4/ε4 was only observed in the *z*-score-derived MCI and pre-MCI SCD groups. Among the 94 individuals with MCI diagnosed using clinical criteria, only 34 (36.2%) were accurately diagnosed, whereas the other 26 (27.7%) had normal cognitive function. The remaining 34 (36.2%) individuals were diagnosed with pre-MCI SCD using objective criteria (Table 1 and Figure 1A). Among the 97 individuals with pre-MCI SCD diagnosed using clinical criteria, only 27 (27.8%) were accurately diagnosed; 30 (30.9%) individuals had MCI, and the remaining 40 (41.24%) had normal cognitive function as determined by objective criteria (Table 1 and Figure 1A). The *z*-score-derived pre-MCI SCD group showed the highest prevalence of PPF, whereas the *z*-score-derived MCI group exhibited the highest prevalence of PF. The *z*-score-derived normal group had a significantly higher prevalence of PF and PPF than the robust normal control group. Only 68.8% of the individuals with *z*-score-derived MCI had PRCF and 72.1% of the individuals with *z*-score-derived pre-MCI SCD had RCF (Table 1).

**TABLE 1:** Demographic, neuropsychological, and clinical characteristics [medians and interquartile ranges (Q25–Q75) for continuous variables and absolute numbers or percentages for categorical variables] of experimental samples after reclassification by adopting objective cognitive assessment (neuropsychological test *z*-scores) criteria and for the robust normal control group.

	*Z*-scores derived MCI (*n* = 64)	*Z*-scores derived pre MCI SCD (*n* = 61)	*Z*-scores derived normal (*n* = 66)	Robust normal (*n* = 144)	χ ^2^	*p*
**Demographics**						
Age (years)	75.00 (69.25, 81.00)^b^	72.00 (67.00, 78.00)	71.00 (66.75, 76.25)	72.00 (67.00, 79.75)	5.081	0.166
Education (years)	9.00 (8.00, 12.00)^a,b^	9.00 (9.00, 12.50)^a^	12.00 (9.00, 14.00)^a^	12.00 (9.00, 16.00)	32.492	0.000
Gender (% male)	29 (45.313%)	19 (31.148%)	30 (45.50%)	72 (50.00%)	6.392	0.094
Apoe ε4/ε4 frequency (*n* = 184)	38/64	38/61	40/66	68/144	13.409	0.037
0	32	30	27	62		
1	5	7	13	6		
2	1	1	0	0		
**Clinical stratification**						
MCI (*n* = 94)	34	34	26	–	–	–
Pre-MCI SCD (*n* = 97)	30	27	40	–	–	–
**Physical frailty**					37.488	0.000
Without PF or PPF	20 (31.3%)	16 (26.2%)	24 (36.4%)	78 (54.167%)		
PPF	26 (40.6%)	38 (62.3%)	36 (54.5%)	59 (40.972%)		
PF	18 (28.1%)	7 (11.5%)	6 (9.1%)	7 (4.861%)		
**Cognitive frailty**					441.183	0.000
Without CF	20 (31.3%)	16 (26.2%)	66 (100%)	144 (100%)		
RCF	–	45 (73.8%)	–	–		
PRCF	44 (68.8%)	–	–	–		
**Objective measures (raw)**						
TMT. A	78.00 (59.00, 121.00)^a,b,c^	54.00 (41.00, 71.50)^a,b^	43.00 (36.00, 59.00)	42.00 (35.00,52.00)	77.000	0.000
TMT. B	118.00 (86.25, 192.50)^a,b,c^	74.00 (60.50, 100.50)^a^	71.50 (58.00, 90.25)^a^	65.00 (52.00, 79.75)	63.926	0.000
HVLT-R delayed recall	2.00 (0.00, 3.50)^a,b,c^	3.00 (1.00, 5.00)^a,b^	5.00 (4.00, 7.00)	6.00 (4.00, 8.00)	90.794	0.000
HVLT-R recognition	10.00 (7.50, 11.00)^a,b^	10.00 (9.00, 11.00)^a,b^	11.00 (10.00, 12.00)	11 (10.00, 12.00)	35.883	0.000
Learning slope	1.00 (0.50, 1.333)^a,b^	1.00 (0.333, 1.00)^a,b^	1.333 (1.00, 1.667)	133 (1.00, 1.67)	39.026	0.000
Intrusion errors	3.00 (1.00, 6.00)	4.00 (1.25, 6.00)	3.00 (2.00, 4.750)	3.00 (1.00, 5.00)	3.312	0.346
Retroactive interference	0.600 (0.330, 0.817)^a^	0.500 (0.298, 0.788)^a,b^	0.667 (0.500, 0.871)	0.75 (0.58, 0.88)	24.456	0.000
BNT	23.00 (20.00, 25.00)^a,b,c^	25.00 (22.75, 27.00)^a,b^	26.000 (25.00, 28.00)^a^	28.00 (26.00, 29.00)	97.207	0.000
Animal fluency	12.00 (10.00, 14.00)^a,b,c^	14.00 (12.00, 17.00)^a,b^	16.50 (15.00, 19.00)^a^	18.00 (16.00, 21.00)	107.188	0.000
Digital span forward	6.00 (5.00, 7.00)^a,b^	6.00 (5.00, 7.00)^a,b^	7.00 (6.00, 8.00)	7.00 (6.00, 8.00)	29.697	0.000
Digital span backward	4.00 (3.00, 4.00)^a,b^	4.00 (3.00, 5.00)^a,b^	4.00 (3.00, 5.00)	4.00 (4.00, 5.00)	18.402	0.000
Digital symbol	22.00 (16.25, 30.00)^a,b^	27.00 (20.50, 35.00)^a,b^	31.00 (23.50, 37.00)	33.00 (27.00, 39.75)	39.047	0.000
**Clinical measures**						
MMSE	26.00 (25.00, 28.00)^a,b^	26.00 (25.00, 27.50)^a,b^	27.00 (26.00, 28.75)^a^	28.00 (27.00, 29.00)	78.825	0.000
CDR = 0.000/0.5 (% = 0.5)	54/6 (10%)	54/1 (1.8%)	63/1 (1.6%)	125/0.000 (0.000)	16.519	0.001
ADAS Cog	20.70 (13.670, 25.330)^a,b,c^	16.660 (13.668, 21.000)^a,b^	13.660 (10.00, 17.340)^a^	10.83 (7.31, 16.00)	60.689	0.000
ADAS Non-Cog	3.00 (1.00, 6.00)^a,b^	2.00 (1.00, 4.00)^a^	2.00 (0.000, 4.00)	2.00 (0.25, 3.00)	14.691	0.002
GDS	3.00 (2.00, 6.00)^a^	4.00 (2.50, 7.00)^a^	3.00 (2.00, 6.00)^a^	2.00 (1.00, 5.00)	20.239	0.000
SCD-Q, MyCog	10.50 (5.25, 13.00)^a^	10.00 (6.50, 12.50)^a^	9.00 (6.75, 13.00)^a^	3.000 (1.00, 4.00)	139.046	0.000
Function *Q*	1.00 (0.00, 6.00)^a,b^	1.00 (0.00, 3.00)^a,b^	0.00 (0.00, 1.00)	0.00 (0.00, 1.00)	28.484	0.000
NPI-Q score	0.00 (0.00, 2.00)^a^	1.00 (0.00, 3.00)^a^	0.00 (0.00, 3.00)^a^	0.00 (0.00, 1.00)	16.362	0.001

*^*a*^Significantly different from robust normal control, ^*b*^significantly different from Z-scores derived normal control, ^*c*^significantly different from Z-scores derived pre-MCI SCD. Number of subjects with data. Qi, i^*th*^ quantile.*

*BNT, Boston Naming Test; TMT. Part A and B, Trail Making Test A and B; HVLT-R, the Hopkins Verbal Learning Test-Revised; MMSE, the Mini Mental State evaluation; CDR, Clinical Dementia Rating; ADAS Cog, cognitive subscale of the Alzheimer’s Disease Assessment Scale; ADAS Non-Cog, non-cognitive subscale of the Alzheimer’s Disease Assessment Scale; GDS, the Geriatric Depression Scale; SCD-Q, subjective cognitive decline questionnaire; FAQ, Functional Assessment Questionnaire; NPI-Q, neuropsychiatric inventory questionnaire; PRCF, Potentially reversible cognitive frailty; RCF, Reversible cognitive frailty; PF, Physical frailty; PPF, Physical pre-frailty*.

All raw scores of objective measures, excluding the process scores of intrusion errors, revealed significant differences among the four subgroups (Table 1). However, clinical measures demonstrated less ability to discriminate CI severity compared with objective raw scores. Only ADAS-Cog scores were significantly different between the *z*-score-derived normal or robust normal groups and the *z*-score-derived MCI and pre-MCI SCD groups and could differentiate between the *z*-score-derived MCI and pre-MCI SCD groups as well as between the *z*-score-derived normal and robust normal groups (Table 1). The MMSE scores were significantly higher in the *z*-score-derived normal or robust normal groups than in the *z*-score-derived MCI and pre-MCI SCD groups. However, the MMSE scores did not significantly differ between the *z*-score-derived MCI and pre-MCI SCD groups. ADAS Non-Cog scores were significantly different between the *z*-score-derived normal and pre-MCI SCD groups and between the pre-MCI SCD and MCI groups. The CDR scores and SCD-Q MyCog scores were not significantly different among the *z*-score-derived normal, MCI, and pre-MCI SCD groups.

After adjusting for demographic factors, the scores of objective cognitive measures, including TMT A, TMT B, HVLT-R recognition, learning slope, retroactive interference, digit span forward, digit span backward, and digit symbol, were significantly different between the *z*-score-derived normal or robust normal and the *z*-score-derived MCI and pre-MCI SCD groups and not between the *z*-score-derived normal or robust normal groups (Table 2). Only the scores of intrusion errors showed no significant difference among the four groups. The discriminating ability of clinical measures for CI evidently improved after adjusting for age, sex, and education level. The MMSE, ADAS-Cog, and SCD-Q MyCog scores significantly differed between the *z*-score-derived normal or robust normal and the *z*-score-derived MCI and pre-MCI SCD groups as well as between the *z*-score-derived normal and robust normal groups (Table 2). Compared with the robust normal control, the scores of the non-cognitive measures ADAS Non-Cog, GDS, FAQ, and NPI-Q were significantly higher in the *z*-score-derived MCI and pre-MCI SCD groups. In addition, the GDS and NPI-Q scores were significantly or marginally high in the *z*-score-derived normal group.

**TABLE 2:** Results from nominal regression analyses that evaluated the difference of cognitive stratification by clinical or objective measures after adjusting by sex, age, and education level (the reference category is robust normal).

	*Z*-scores derived MCI			*Z*-scores derived pre-MCI SCD			*Z*-scores derived normal		
			
	*B* (standard error)	OR(95%CI)	*p*	*B* (standard error)	OR(95%CI)	*p*	*B* (standard error)	OR(95%CI)	*p*
**Objective measures**									
TMT A	0.067 (0.010)	1.069 (1.048, 1.091)	0.000	0.047 (0.010)	1.048 (1.028, 1.068)	0.000	0.017 (0.010)	1.017 (0.997, 1.038)	0.094
TMT B	0.038 (0.006)	1.039 (1.026, 1.052)	0.000	0.012 (0.006)	1.012 (1.000, 1.025)	0.050	0.010 (0.006)	1.010 (0.998, 1.023)	0.092
HVLT-R delayed recall	−0.721(0.098)	0.486 (0.401, 0.589)	0.000	−0.551(0.087)	0.576 (0.486, 0.683)	0.000	−0.141(0.069)	0.868 (0.759, 0.994)	0.040
HVLT-R recognition	−0.560(0.107)	0.571 (0.463, 0.705)	0.000	−0.396(0.108)	0.673 (0.545, 0.831)	0.000	−0.054(0.121)	0.947 (0.748, 1.200)	0.654
Learning slope	−0.856(0.283)	0.425 (0.244, 0.740)	0.002	−1.198(0.282)	0.302 (0.174, 0.524)	0.000	0.227 (0.272)	1.254 (0.737, 2.136)	0.404
Intrusion errors	0.078 (0.056)	1.082 (0.969, 1.207)	0.162	0.090 (0.053)	1.094 (0.987, 1.213)	0.089	0.015 (0.055)	1.015 (0.911, 1.131)	0.786
Retroactive interference	−0.746(0.439)	0.474 (0.201, 1.121)	0.089	−1.043(0.489)	0.352 (0.135, 0.919)	0.033	−0.518(0.425)	0.596 (0.259, 1.371)	0.223
BNT	−0.609(0.081)	0.544 (0.464, 0.638)	0.000	−0.405(0.075)	0.667 (0.576, 0.772)	0.000	−0.257(0.071)	0.774 (0.673, 0.889)	0.000
Animal fluency	−0.471(0.063)	0.624 (0.551, 0.706)	0.000	−0.297(0.054)	0.743 (0.669, 0.826)	0.000	−0.129(0.043)	0.879 (0.808, 0.956)	0.003
Digital span forward	−0.349(0.118)	0.705 (0.559, 0.889)	0.003	−0.240(0.113)	0.787 (0.630, 0.982)	0.034	−0.059(0.111)	0.943 (0.759, 1.171)	0.595
Digital span backward	−0.412(0.176)	0.663 (0.469, 0.935)	0.019	−0.223(0.159)	0.800 (0.587, 1.092)	0.160	0.082 (0.139)	1.085 (0.827, 1.424)	0.555
Digital symbol	−0.101(0.023)	0.904 (0.863, 0.946)	0.000	−0.072(0.021)	0.931 (0.893, 0.970)	0.001	−0.024(0.018)	0.976 (0.942, 1.011)	0.182
**Clinical measures**									
MMSE	−0.687(0.111)	0.503 (0.405, 0.626)	0.000	−0.707(0.110)	0.493 (0.397, 0.612)	0.000	−0.524(0.104)	0.592 (0.483, 0.726)	0.000
ADAS Cog	0.196 (0.031)	1.217 (1.146, 1.293)	0.000	0.158 (0.029)	1.172 (1.106, 1.241)	0.000	0.062 (0.028)	1.064 (1.008, 1.124)	0.024
ADAS Non-Cog	0.189 (0.057)	1.208 (1.081, 1.350)	0.001	0.072 (0.062)	1.075 (0.952, 1.214)	0.244	0.051 (0.061)	1.053 (0.934, 1.186)	0.399
GDS	0.137 (0.055)	1.147 (1.030, 1.277)	0.012	0.161 (0.052)	1.174 (1.060, 1.301)	0.002	0.103 (0.051)	1.109 (1.002, 1.226)	0.045
SCD-Q, MyCog	0.485 (0.062)	1.625 (1.440, 1.833)	0.000	0.491 (0.062)	1.633 (1.448, 1.843)	0.000	0.510 (0.061)	1.665 (1.476, 1.877)	0.000
FAQ sore	0.418 (0.091)	1.519 (1.271, 1.815)	0.000	0.306 (0.092)	1.359 (1.135, 1.626)	0.001	0.067 (0.115)	1.069 (0.854, 1.339)	0.559
NPI-Q score	0.210 (0.078)	1.234 (1.060, 1.437)	0.007	0.248 (0.076)	1.281 (1.103, 1.488)	0.001	0.142 (0.084)	1.153 (0.978, 1.358)	0.089

*TMT. Part A and B, Trail Making Test A and B; HVLT-R, the Hopkins Verbal Learning Test-Revised; BNT, Boston Naming Test; MMSE, the Mini Mental State evaluation; ADAS Cog, cognitive subscale of the Alzheimer’s Disease Assessment Scale; ADAS Non-Cog, non-cognitive subscale of the Alzheimer’s Disease Assessment Scale; GDS, the Geriatric Depression Scale; SCD-Q, subjective cognitive decline questionnaire; FAQ, Functional Assessment Questionnaire; NPI-Q, neuropsychiatric inventory questionnaire.*

### Comparison Between Clinical and Objective Assessments of CF

Characteristics of CF, PF/PPF only, CI only (*z*-scored derived pre-MCI SCD/MCI), and normal (no PF/PPF and CI) subgroups reclassified according to the Fried PF/PPF, objective cognitive, and CF criteria are presented in Table 3. There were significant differences in age and education level but not in sex. The frequency of ApoE ε4 was lower in the normal group (12.96%) than in other subgroups, and individuals with ApoE ε4/ε4 were only observed in the CF and CI groups.

**TABLE 3:** Demographic, neuropsychological, and clinical characteristics (medians and interquartile ranges [Q25–Q75] for continuous variables, and absolute numbers or percentages for categorical variables) of experimental samples in four stratifications after reclassification by adopting objective cognitive assessment (neuropsychological test *z*-scores) combined with Fried PF score criteria.

	CF (RCF + PRCF) (*n* = 89)	Only PF/PPF (*N* = 108)	Only *z*-score-derived pre-MCI SCD/MCI (Pre-MCI SCD + MCI) (*N* = 36)	Normal (no PF/PPF and cognitive impairment) (*N* = 102)	χ ^2^	*p*
**Demographics**						
Age (years)	75.00(69.00, 86.00)	73.00(68.00, 80.00)	70.00(65.00, 75.00)^a,b^	71.00(65.00, 76.25)^a,b^	19.782	0.000
Education (years)	9.00(8.00, 13.50)	12.00(9.00, 16.00)^a^	9.00(9.00, 12.00)^b^	12.00(9.00, 15.00)^a,c^	26.092	0.000
Gender (% male)	33.00(37.08%)	55.00(50.93%)	15.00(41.67%)	47.00(46.08%)	3.789	0.285
Apoe ε4/ε4 frequency (*n* = 184)	52/89	54/108	24/36	54/102	5.389	0.495
0	43	42	19	47		
1	8	12	4	7		
2	1	0	1	0		
**Clinical stratification**						
MCI (*n* = 94)	25 + 22	16	9 + 12	10	−	−
Pre-MCI SCD (*n* = 97)	20 + 22	26	7 + 8	14	−	−
**Objective stratification**					342.654	0.000
*Z*-scores derived MCI	44	0	20	0		
*Z*-scores derived pre MCI SCD	45	0	16	0		
*Z*-scores derived normal	0	42	0	24		
Robust normal	0	66	0	78		
**Objective measures**						
TMT. A	56.50(41.75, 84.25)	44.00(37.00, 58.00)^a^	59.50(42.75, 99.75)^b^	40.50(33.75, 50.00)^a,b,c^	66.307	0.000
TMT. B	74.5(58.75, 108)	68.00(57.50, 83.00)^a^	79.00(67.00, 146.00)^b^	65.00(51.00, 84.00)^a,c^	40.333	0.090
HVLT-R delayed recall	3.41(1, 5)	5.00(4.00, 7.00)^a^	2.00(0.00, 4.00)^b^	6.00(4.00, 8.00)^a,b,c^	93.872	0.000
HVLT-R recognition	10.5(9, 11)	11.00(10.00, 12.00)^a^	9.50(9.00, 10.00)^b^	11.00(10.00, 12.00)^a,c^	36.627	0.000
Learning slope	1(0.33, 1.33)	1.33(1.00, 1.67)^a^	1.00(0.33, 1.33)^b^	1.33(1.00, 1.67)^a,c^	38.097	0.000
Intrusion errors	4(2, 6.75)	3.00(1.00, 5.00)	3.00(1.00, 5.00)	3.00(2.00, 5.00)	2.513	0.473
Retroactive interference	0.5(0.29, 0.66)	0.75(0.50, 0.88)^a^	0.59(0.34, 0.80)^b^	0.71(0.60, 0.86)^a,c^	20.922	0.000
BNT	25.00(22.00, 27.00)	27.00(25.00, 28.00)^a^	24.00(23.00, 25.75)^b^	27.50(26.00, 29.00)^a,c,^	78.540	0.000
Animal fluency	14(11, 17)	18.00(15.00, 19.25)^a^	13.50(12.00, 15.75)^b^	18.00(15.00, 21.00)^a,c^	92.347	0.000
Digital span forward	6(5, 7)	7.00(6.00, 8.00)^a^	6.00(5.00, 7.00)^b^	7.00(6.00, 8.00)^a,c^	27.074	0.000
Digital span backward	4(3, 5)	4.00(4.00, 5.00)^a^	4.00(3.00, 4.00)^b^	4.00(4.00, 5.00)^*c*^	17.758	0.000
Digital symbol	26(19.5, 32)	33.00(24.00, 36.50)^a^	30.00(23.25, 38.25)^a^	33.00(28.25, 41.00)^a,c^	44.288	0.000
**Clinical measures**						
MMSE	26.00(25.00, 27.00)	28.00(27.00,29.00)^a^	25.00(26.00, 28.00)^b^	28.00(27.00, 29.00)^a,b,c^	59.598	0.000
CDR = 0.000/0.5 (% = 0.5)	75.00/6.00(7.41%)	99.00/1.00(1.00%)	32/1(3.03%)	88.00/0.00(0.00%)	10.579	0.0014
ADAS Cog	17.33(13.92, 20.75)	13.00(8.00, 17.00)^a^	16.48(9.75, 22.47)^b^	10.83(7.32, 15.66)^a,c^	62.342	0.000
ADAS Non-Cog	3(2, 4)	2.00(1.00, 4.00)^a^	2.00(1.00, 4.00)	1.00(0.00, 3.00)^a^	18.732	0.059
GDS	4(2.5, 7)	3.00(1.00, 6.00)^a^	3.00(2.00, 6.00)	2.00(1.00, 4.00)^a,b^	19.924	0.000
SCD-Q, MyCog	10(6, 12)	5.00(3.00, 8.00)^a^	8.00(5.00, 12.75)^b^	3.00(1.00, 5.00)^a,b,c^	72.395	0.000
Function Q	2(2, 5.5)	0.00(0.00, 1.00)^a^	0.00(0.00, 1.00)^a^	0.00(0.00, 1.00)^a^	42.160	0.000
NPI-Q score	0(0, 2.5)	0.00(0.00, 2.00)^a^	0(0.00, 2.00)	0.00(0.00, 1.00)^a^	13.885	0.003

**^*a*^Significantly different from CF, ^*b*^significantly different from only PF/PPF, ^*c*^significantly different from pre-MCI SCD/MCI. N, Number of subjects. Qi, i^*th*^ quantile. SCD, subjective cognitive decline; MCI, mild cognitive impairment; CF, cognitive frailty; PF, physical frailty; PPF, physical pre-frailty; BNT, Boston Naming Test; TMT. A and B, Trail Making Test A and B; HVLT-R, the Hopkins Verbal Learning Test-Revised; MMSE, the Mini Mental State evaluation; CDR, Clinical Dementia Rating; ADAS Cog, cognitive subscale of the Alzheimer’s Disease Assessment Scale; ADAS Non-Cog, non-cognitive subscale of the Alzheimer’s Disease Assessment Scale; GDS, the Geriatric Depression Scale; SCD-Q, subjective cognitive decline questionnaire; FAQ, Functional Assessment Questionnaire; NPI-Q, neuropsychiatric inventory questionnaire*.*

When the clinical cognitive criteria were integrated into the CF criteria, 63 of 94 individuals were diagnosed with PRCF and 68 of 97 were diagnosed with RCF (Table 3). However, when objective cognitive criteria replaced the clinical criteria in the CF criteria, only 22 of 63 (34.9%) individuals were diagnosed with PRCF. Conversely, 25 (39.7%) individuals were diagnosed with RCF, and 16 (25.4%) were cognitively normal. Similarly, only 20 of 68 (29.4%) individuals were diagnosed with RCF. Of the remaining individuals, 22 (32.4%) were diagnosed with PRCF, and 26 (38.2%) were cognitively normal (Figure 1B).

The raw scores of objective tests demonstrated better discriminative ability than clinical cognitive assessment scores. Apart from the scores of intrusion errors, the scores of the other nine objective tests could accurately discriminate the cognitive status of CF, PF/PPF only, pre-MCI SCD/MCI only, and normal groups; only TMT A and HVLT-R delayed recall scores could not accurately discriminate the cognitive status between PF/PPF only and normal groups. However, the clinical MMSE and SCD-Q MyCog scores could accurately discriminate the cognitive status among CI groups (CF and pre-MCI SCD/MCI only groups), PF/PPF only, and normal groups (Table 3). Furthermore, only ADAS-Cog scores could accurately discriminate the cognitive status of the four groups.

After covariate adjustment, the discriminating ability of objective and clinical measures for CI showed distinct improvement. Apart from the scores of the two processes (intrusion errors and retroactive interference) and the measure digit span backward, the other objective measures for CI diagnosis could accurately discriminate the cognitive status of CF, PF/PPF only, pre-MCI SCD/MCI only, and normal groups (Table 4). The MMSE, SCD-Q MyCog, and ADAS-Cog scores could accurately discriminate the cognitive status of the four abovementioned groups (Table 4). Compared with the CF group, the scores of non-cognitive measures were significantly lower in the other three groups.

**TABLE 4:** Results from nominal regression analyses that evaluated the difference of cognitive stratification by clinical or objective measures after adjusting by sex, age, and education level (the reference category is cognitive frailty).

	Only PF/PPF			Only pre-MCI SCD or MCI			Normal		
			
	*B* (standard error)	OR(95%CI)	*p*	*B* (standard error)	OR(95%CI)	*P*	*B* (standard error)	OR(95%CI)	*p*
**Objective measures**									
TMT A	−0.045(0.009)	0.956 (0.939, 0.972)	0.000	−0.001(0.006)	0.999 (0.988, 1.011)	0.897	−0.054(0.010)	0.948 (0.929, 0.967)	0.000
TMT B	−0.021(0.005)	0.979 (0.969, 0.989)	0.000	0.002 (0.004)	1.002 (0.995, 1.010)	0.546	−0.020(0.005)	0.981 (0.971, 0.991)	0.000
HVLT-R delayed recall	0.485 (0.083)	1.624 (1.381, 1.910)	0.000	−0.171(0.105)	0.843 (0.686, 1.036)	0.104	0.586 (0.087)	1.796 (1.514, 2.132)	0.000
HVLT-R recognition	0.424 (0.106)	1.528 (1.242, 1.880)	0.000	−0.079(0.090)	0.924 (0.774, 1.103)	0.379	0.429 (0.110)	1.536 (1.239, 1.905)	0.000
Learning slope	1.086 (0.280)	2.961 (1.712, 5.123)	0.000	0.220 (0.304)	1.246 (0.687, 2.259)	0.470	1.299 (0.289)	3.667 (2.081, 6.462)	0.000
Intrusion errors	−0.097(0.053)	0.907 (0.818, 1.006)	0.065	−0.058(0.067)	0.944 (0.827, 1.076)	0.386	−0.094(0.053)	0.910 (0.820, 1.011)	0.078
Retroactive interference	0.573 (0.401)	1.773 (0.808, 3.888)	0.153	−0.521(0.633)	0.594 (0.172, 2.053)	0.410	0.605 (0.410)	1.831 (0.821, 4.088)	0.140
BNT	0.359 (0.065)	1.432 (1.260, 1.628)	0.000	0.030 (0.067)	1.031 (0.905, 1.175)	0.648	0.430 (0.072)	1.537 (1.335, 1.769)	0.000
Animal fluency	0.327 (0.052)	1.386 (1.251, 1.537)	0.000	0.064 (0.058)	1.066 (0.952, 1.193)	0.271	0.361 (0.054)	1.435 (1.290, 1.596)	0.003
Digital span forward	0.339 (0.113)	1.404 (1.125, 1.750)	0.003	0.070 (0.143)	1.073 (0.811, 1.420)	0.622	0.237 (0.114)	1.268 (1.014, 1.585)	0.037
Digital span backward	0.370 (0.154)	1.448 (1.070, 1.959)	0.017	−0.203(0.220)	0.816 (0.530, 1.257)	0.356	0.170 (0.159)	1.185 (0.867, 1.620)	0.287
Digital symbol	0.087 (0.022)	1.091 (1.045, 1.138)	0.000	0.055 (0.027)	1.056 (1.002, 1.113)	0.042	0.105 (0.022)	1.111 (1.063, 1.160)	0.000
**Clinical measures**									
MMSE	0.388 (0.093)	1.474 (1.227, 1.770)	0.000	0.007 (0.110)	1.007 (0.812, 1.249)	0.949	0.558 (0.103)	1.747 (1.428, 2.137)	0.000
ADAS Cog	−0.164(0.029)	0.849 (0.802, 0.898)	0.000	−0.061(0.032)	0.940 (0.883, 1.002)	0.056	−0.186(0.031)	0.830 (0.782, 0.882)	0.000
ADAS Non-Cog	−0.111(0.051)	0.895 (0.809, 0.990)	0.031	−0.158(0.076)	0.854 (0.736, 0.991)	0.037	−0.232(0.064)	0.793 (0.699, 0.899)	0.000
GDS	−0.087(0.048)	0.916 (0.834, 1.007)	0.070	−0.145(0.069)	0.865 (0.756, 0.990)	0.035	−0.234(0.057)	0.792 (0.708, 0.884)	0.000
SCD-Q, MyCog	−0.118(0.032)	0.889 (0.835, 0.945)	0.000	−0.018(0.038)	0.983 (0.912, 1.059)	0.645	−0.255(0.041)	0.775 (0.716, 0.840)	0.000
FAQ sore	−0.328(0.081)	0.721 (0.614, 0.845)	0.000	−0.228(0.092)	0.796 (0.6655, 0.953)	0.013	−0.480(0.117)	0.618 (0.492, 0.777)	0.000
NPI-Q score	−0.142(0.062)	0.867 (0.768, 0.979)	0.021	−0.137(0.090)	0.872 (0.730, 1.040)	0.128	−0.307(0.093)	0.735 (0.613, 0.883)	0.001

*TMT. Part A and B, Trail Making Test A and B; HVLT-R, the Hopkins Verbal Learning Test-Revised; BNT, Boston Naming Test; MMSE, the Mini Mental State evaluation; ADAS Cog, cognitive subscale of the Alzheimer’s Disease Assessment Scale; ADAS Non-Cog, non-cognitive subscale of the Alzheimer’s Disease Assessment Scale; GDS, the Geriatric Depression Scale; SCD-Q, subjective cognitive decline questionnaire; FAQ, Functional Assessment Questionnaire; NPI-Q, neuropsychiatric inventory questionnaire.*

### Comparison Between Clinical and Objective Assessments of CF Subtypes

The CF and pre-MCI SCD/MCI only groups in Table 3 were divided into two subtypes according to CI severity (Table 5). Significant differences were noted in RCF, PRCF, pre-MCI SCD only, MCI only, and normal groups with respect to age and education level but not sex. ApoE ε4/ε4 was only observed in the RCF and MCI only groups.

**TABLE 5:** Demographic, neuropsychological, and clinical characteristics (medians and interquartile ranges [Q25–Q75] for continuous variables, and absolute numbers or percentages for categorical variables) of experimental samples in four stratifications after reclassification by adopting objective cognitive assessment (neuropsychological test *z*-scores) combined with Fried PF score criteria.

	RCF(*n* = 45)	PRCF(*n* = 44)	Only pre- MCI SCD (*n* = 16)	Only MCI (*n* = 20)	Normal (*N* = 102)	χ ^2^	*p*
**Demographics**							
Age (years)	75(69.00, 78.00)	77.00(72.00, 81.75)	70.00(64.00, 73.50)^a,b^	71.50(67.00, 75.75)^b^	71.00(65.00, 76.00)^a,b^	21.729	0.00
Education (years)	9.00(8.00, 13.50)	9.00 (8.00, 12.00)	11.50(9.00, 12.75)	9.00(6.75, 12.00)	12.00(9.00, 15.00)^a,b,d^	22.088	0.00
Gender (% male)	12.00(26.67%)	21(47.73%)	7(43.75%)	8(40%)	47(46.08%)	5.840	0.211
Apoe ε4/ε4 frequency (*n* = 130)						7.027	0.543
0	20	23	10	9	47		
1	5	3	2	2	7		
2	1	0	0	1	0		
**Objective measures**							
TMT. A	56.50(41.75,84.25)	81.00(61.00, 124.00)^a^	50.50(35.75, 66.50)^b^	65.50(54.25, 119.50)^*c*^	40.00(33.50, 50.00)^a,b,c,d^	71.045	0.00
TMT. B	74.50(58.75, 108.00)	124.50(91.75, 226.00)^a^	71.00(66.00, 93.00)^b^	100.00(68.75, 180.00)^a,c^	65.00(51.00, 85.50)^a,b,d^	53.774	0.00
HVLT-R delayed recall	4.00(1.00, 5.00)	2.00(0.00, 3.00)^a^	2.00(1.00, 3.75)	1.50(0.00, 4.75)	6.00(4.75, 8.00)^a,b,c,d^	83.918	0.00
HVLT-R recognition	10.50(9.00, 11.00)	10.00(6.50, 11.00)	10.00(9.00, 10.75)	9.00(8.25, 10.00)^a^	11.00(10.00, 12.00)^a,b,c,d^	32.961	0.00
Learning slope	1.00(0.33, 1.33)	0.67(0.50, 1.33)	0.67(0.33, 1.00)	1.00(0.42, 1.67)	1.33(1.00, 1.67)^a,b,c^	34.024	0.00
Intrusion errors	4.00(2.00, 6.75)	3.00(1.00, 7.00)	3.50(0.25, 5.00)	2.50(1.25, 5.75)	3.00(2.00, 5.00)	3.038	0.55
Retroactive interference	0.50(0.29, 0.66)	0.60(0.27, 0.86)	0.65(0.33, 0.96)	0.59(0.38, 0.75)	0.72(0.62, 0.86)^a,b,d^	23.350	0.00
BNT	25.00(22.00, 27.00)	22.00(19.00, 24.50)^a^	25.00(24.00, 27.00)^b^	24.00(22.00, 25.00)	28.00(26.00, 29.00)^a,b,c,d^	75.301	0.00
Animal fluency	14.00(11.00, 17.00)	12.00(9.00, 14.00)^a^	14.50(12.00, 16.75)^b^	13.00(11.00, 15.00)	18.00(15.00, 21.00)^a,b,c,d^	77.079	0.00
Digital span forward	6.00(5.00, 7.00)	5.00(4.25, 7.00)	6.00(5.00, 7.00)	6.00(5.00, 7.00)	7.00(5.00, 8.00)^a,b^	18.536	0.001
Digital span backward	4.00(3.00, 5.00)	4.00(3.00, 4.00)^a^	3.00(3.00, 4.00)	4.00(4.00, 4.00)^b,c^	4.00(4.00, 5.00)^b,c^	19.281	0.001
Digital symbol	26.00(19.50, 32.00)	19.00(16.00, 27.50)^a^	32.00(26.00, 38.75)^b^	29.50(19.25, 36.75)^b^	33.00(28.00, 41.00)^a,b^	46.111	0.00
**Clinical measures**							
MMSE	26.00(25.00,27.00)	26.00(25.00, 28.00)	26.00(25.00, 28.00)	26.00(25.00, 28.00)	28.00(27.00, 29.00)^a,b,c,d^	52.055	0.00
CDR = 0.000/0.5(% = 0.5)	41/1(2.38%)	35/5(12.5%)	13/0(0.00%)	19/1(5.00%)	88/0(0.00%)	13.739	0.008
ADAS Cog	17.33(13.92, 20.745)	21.66(15.00, 29.99)^a^	16.15(10.83, 21.68)^b^	18.00(9.41, 22.66)^b^	10.30(7.00, 15.66)^a,b,c,d^	55.900	0.00
ADAS Non-Cog	3.00(2.00, 4.00)	4.00(1.00, 8.00)	2.00(0.25, 3.00)^b^	2.00(1.00, 4.75)	1.00(0.00, 3.00)^a,b^	20.966	0.00
GDS	4.00(2.50, 7.00)	4.00(2.00, 7.00)	4.00(2.25, 6.75)	2.00(1.00, 5.00)^a,b,c^	2.00(1.00, 4.00)^a,b,c^	25.386	0.00
SCD-Q, MyCog	10.00(6.00, 12.00)	11.50(7.00, 13.75)	10.50(7.00, 13.00)	7.50(3.25, 11.50)	3.00(1.00, 5.00)^a,b,c,d^	67.197	0.00
Function *Q*	2.00(0.00, 5.00)	2.50(0.00, 9.75)	0.00(0.00, 1.00)^a,b^	0.00(0.00, 1.75)^b^	0.00(0.00, 1.00)^a,b^	38.876	0.000
NPI-Q score	0.00(0.00, 2.00)	2.00(0.00, 4.00)	1.00(0.00, 3.00)	0.00(0.00, 0.00)^a,b,c^	0.00(0.00, 1.00)^a,b,c^	21.806	0.00

**^*a*^Significantly different from RCF, ^*b*^significantly different from PRCF, ^*c*^significantly different from only pre-MCI SCD, ^*d*^significantly different from only MCI. N, number of subjects. Qi, i^*th*^ quantile. SCD, subjective cognitive decline; MCI, mild cognitive impairment; CF, cognitive frailty; PF, Physical frailty; PPF, Physical pre-frailty; BNT, Boston Naming Test; TMT. A and B, Trail Making Test A and B; HVLT-R, the Hopkins Verbal Learning Test-Revised; MMSE, the Mini Mental State evaluation; CDR, Clinical Dementia Rating; ADAS Cog, cognitive subscale of the Alzheimer’s Disease Assessment Scale; ADAS Non-Cog, non-cognitive subscale of the Alzheimer’s Disease Assessment Scale; GDS, the Geriatric Depression Scale; SCD-Q, subjective cognitive decline questionnaire; FAQ, Functional Assessment Questionnaire; NPI-Q, neuropsychiatric inventory questionnaire*.*

Five of six total raw scores (excluding HVLT-R recognition) for objective cognitive assessment were significantly different between the RCF and PRCF groups, and two of six total raw scores were significantly different in the pre-MCI SCD only and MCI only groups. However, two scores (MMSE and SCD-Q MyCog) for clinical cognitive assessment were not significantly different between the RCF and PRCF groups and between the pre-MCI SCD only and MCI only groups.

After covariate adjustment, the six total scores for objective cognition assessment were significantly different between the RCF and PRCF groups (Table 6). Among the six total scores, the TMT A and TMT B scores significantly differed between the RCF and MCI only groups, whereas the HVLT-R delayed recall and HVLT-R recognition scores marginally differed between the RCF and only MCI groups. Among the three process scores, the learning slope scores significantly differed between the RCF and normal groups. Moreover, the digit span backward and digit symbol scores significantly differed between the RCF and PRCF groups but not between the RCF and only MCI groups. However, the clinical MMSE and SCD-Q MyCog scores did not significantly differ between the RCF and PRCF groups and between the RCF and MCI only groups. The ADAS-Cog scores significantly differed between the RCF and PRCF groups but not between the RCF and MCI only groups. The non-cognitive performance scores significantly differed between the normal and other groups. Compared with the RCF group, the ADAS Non-Cog and FAQ scores were significantly higher in the PRCF group, and the GDS and NPI-Q scores were significantly lower in the MCI only group.

**TABLE 6:** Results from nominal regression analyses that evaluated the difference of cognitive stratification by clinical or objective measures after adjusting by sex, age, and education level (the reference category is RCF).

	PRCF			Only pre-MCI SCD			Only MCI			Normal		
				
	*B* (standard error)	OR (95%CI)	*p*	*B* (standard error)	OR (95%CI)	*P*	*B* (standard error)	OR (95%CI)	*p*	*B* (standard error)	OR (95%CI)	*p*
**Objective measures**												
TMT A	0.021 (0.008)	1.021 (1.006, 1.037)	0.006	−0.004(0.012)	0.996 (0.973, 1.021)	0.774	0.017 (0.009)	1.017 (1.000, 1.034)	0.047	−0.041(0.011)	0.960 (0.939, 0.981)	0.000
TMT B	0.031 (0.007)	1.031 (1.016, 1.047)	0.000	0.006 (0.011)	1.006 (0.985, 1.028)	0.573	0.030 (0.008)	1.030 (1.014, 1.046)	0.000	−0.005(0.007)	0.995 (0.981, 1.010)	0.531
HVLT-R delayed recall	−0.251(0.109)	0.778 (0.628, 0.963)	0.021	−0.342(0.156)	0.7106 (0.523, 0.964)	0.028	−0.236(0.136)	0.790 (0.605, 1.031)	0.083	0.479 (0.102)	1.615 (1.322, 1.973)	0.000
HVLT-R recognition	−0.256(0.117)	0.774 (0.615, 0.974)	0.029	−0.218(0.148)	0.804 (0.601, 1.076)	0.142	−0.242(0.135)	0.785 (0.602, 1.024)	0.074	0.397 (0.143)	1.487 (1.123, 1.970)	0.006
Learning slope	0158 (0.363)	1.171 (0.575, 2.386)	0.663	−0.219(0.467)	0.804 (0.322, 2.008)	0.640	0.724 (0.468)	2.062 (0.824, 5.160)	0.122	1.480 (0.364)	4.395 (2.154, 8.968)	0.000
Intrusion errors	0.000 (0.071)	1.000 (0.870, 1.150)	0.996	−0.068(0.099)	0.935 (0.770, 1.135)	0.495	−0.058(0.089)	0.944 (0.792, 1.124)	0.516	−0.090(0.062)	0.914 (0.809, 1.033)	0.150
Retroactive interference	0.549 (0.567)	1.731 (0.569, 5.264)	0.334	0.530 (0.812)	1.698 (0.346, 8.335)	0.514	−0.504(0.853)	0.604 (0.113, 3.216)	0.555	0.924 (0.548)	2.518 (0.861, 7.366)	0.092
BNT	−0.254(0.079)	0.775 (0.664, 0.906)	0.001	−0.044(0.108)	0.957 (0.775, 1.182)	0.685	−0.126(0.094)	0.882 (0.733, 1.061)	0.184	0.350 (0.085)	1.420 (1.202, 1.677)	0.000
Animal fluency	−0.212(0.073)	0.809 (0.701, 0.933)	0.004	−0.009(0.082)	0.991 (0.845, 1.163)	0.915	−0.086(0.081)	0.918 (0.784, 1.075)	0.288	0.212 (0.061)	1.236 (1.097, 1.392)	0.001
Digital span forward	−0.229(0.171)	0.795 (0.569, 1.112)	0.181	−0.031(0.215)	0.970 (0.636, 1.479)	0.886	0.059 (0.205)	1.060 (0.710, 1.584)	0.775	0.137 (0.144)	1.147 (0.866, 1.520)	0.339
Digital span backward	−0.697(0.263)	0.498 (0.298, 0.834)	0.008	−1.106(0.384)	0.331 (0.156, 0.702)	0.004	−0.087(0.278)	0.917 (0.531, 1.583)	0.755	−0.100(0.189)	0.905 (0.624, 1.312)	0.597
Digital symbol	−0.065(0.032)	0.937 (0.881, 0.998)	0.042	0.015 (0.037)	1.015 (0.944, 1.091)	0.686	0.030 (0.035)	1.030 (0.962, 1.103)	0.394	0.071 (0.026)	1.073 (1.020, 1.129)	0.006
**Clinical measures**												
MMSE	0.002 (0.115)	1.002 (0.800, 1.255)	0.985	0.040 (0.166)	1.041 (0.751, 1.441)	0.811	0.042 (0.145)	1.043 (0.785, 1.386)	0.772	0.594 (0.123)	1.811 (1.424, 2.302)	0.000
ADAS Cog	0.066 (0.033)	1.068 (1.001, 1.139)	0.046	−0.025(0.046)	0.976 (0.891, 1.068)	0.591	−0.044(0.043)	0.957 (0.879, 1.042)	0.316	−0.161(0.035)	0.851 (0.794, 0.912)	0.000
ADAS Non-Cog	0.152 (0.072)	1.165 (1.010, 1.342)	0.035	−0.113(0.127)	0.893 (0.696, 1.146)	0.374	−0.054(0.104)	0.948 (0.773, 1.162)	0.604	−0.168(0.081)	0.845 (0.722, 0.991)	0.038
GDS	0.040 (0.071)	1.041 (0.906, 1.195)	0.570	−0.036(0.099)	0.965 (0.795, 1.171)	0.717	−0.251(0.112)	0.778 (0.625, 0.968)	0.025	−0.242(0.071)	0.785 (0.682, 0.902)	0.001
SCD-Q, MyCog	0.037 (0.046)	1.038 (0.949, 1.135)	0.419	0.089 (0.064)	1.094 (0.965, 1.239)	0.161	−0.028(0.058)	0.972 (0.868, 1.090)	0.631	−0.249(0.050)	0.780 (0.707, 0.860)	0.000
FAQ sore	0.120 (0.052)	1.128 (1.018, 1.250)	0.021	−0.426(0.262)	0.653 (0.391, 1.092)	0.104	−0.085(0.092)	0.918 (0.766, 1.101)	0.356	−0.416(0.122)	0.660 (0.519, 0.838)	0.001
NPI-Q score	0.020 (0.058)	1.020 (0.911, 1.142)	0.731	0.059 (0.094)	1.061 (0.882, 1.276)	0.531	−0.533(0.244)	0.587 (0.364, 0.947)	0.029	−0.276(0.100)	0.759 (0.623, 0.923)	0.006

*TMT. Part A and B, Trail Making Test A and B; HVLT-R, the Hopkins Verbal Learning Test-Revised; BNT, Boston Naming Test; MMSE, the Mini Mental State evaluation; ADAS Cog, cognitive subscale of the Alzheimer’s Disease Assessment Scale; ADAS Non-Cog, non-cognitive subscale of the Alzheimer’s Disease Assessment Scale; GDS, the Geriatric Depression Scale; SCD-Q, subjective cognitive decline questionnaire; FAQ, Functional Assessment Questionnaire; NPI-Q, neuropsychiatric inventory questionnaire.*

## Discussion

In this comparative study, we established objective criteria for CI and CF subtypes based on the normative *z*-scores of six neuropsychological test batteries (two memory, two attention or executive, and two language domains) and three process scores of memory domain. According to objective criteria, 191 individuals with pre-MCI SCD or MCI were reclassified into *z*-score-derived pre-MCI SCD, MCI, and *z*-score-derived normal subgroups and compared with the robust normal group. The main findings are as follows:

(1) The normative *z*-scores could improve the differentiation performance for CI subtypes (Tables 1, 2), CF (Tables 3, 4), and CF subtypes (Tables 5, 6) among four different subgroups compared with the raw scores of six neuropsychological test batteries.
(2) The three other neuropsychological test batteries (digit span forward or backward and digit symbol) further confirmed the previous finding.
(3) Among the clinical measures, only ADAS-Cog scores could differentiate the *z*-score-derived normal subgroup from the *z*-score-derived pre-MCI SCD and MCI groups and RCF from the PRCF before and after adjusting for demographic factors.
(4) The raw MMSE scores could differentiate the *z*-score-derived normal from the *z*-score-derived MCI and pre-MCI SCD groups (Table 1) and the CF and pre-MCI SCD/MCI only groups from the PF/PPF only and normal groups (Table 3) but not the *z*-score-derived MCI group from the pre-MCI SCD group (Table 1) and the RCF group from the PRCF group (Table 5).
(5) SCD-Q MyCog raw scores were not significantly different among the *z*-score-derived normal, MCI, and pre-MCI SCD groups (Table 1). However, the scores varied between the CF and pre-MCI SCD/MCI only groups from PF/PPF only and normal groups (Table 3) but not between the RCF and PRCF groups and pre-MCI SCD only and MCI only groups (Table 5).
(6) After adjusting for demographic factors, the MMSE and SCD-Q MyCog scores could differentiate between the *z*-score-derived normal or robust normal and *z*-score-derived MCI and pre-MCI SCD groups, between the *z*-score-derived normal and robust normal groups (Table 2), and between the PF/PPF only and normal groups and the CF and pre-MCI SCD/MCI only groups (Table 4). However, the scores could not distinguish RCF from PRCF and pre-MCI SCD only from MCI (Table 6).
(7) The Non-Cog scores were higher in the CF group than in other subgroups, and the GDS and NPI-Q scores were significantly lower in the MCI only group than in the RCF group.

Although clinical criteria, including the MMSE and SCD-Q MyCog scores, could better differentiate CI or CF from those with normal cognitive function after covariate adjustment, these clinical tools failed to differentiate between the subtypes of CI (pre-MCI SCD and MCI) and CF (RCF and PRCF). The construct of MCI clinical criteria including SCD may be one of the critical factors owing to which covariate adjustment improves the differentiation in some groups but not in others. Therefore, as indicated by our findings, clinical cognitive criteria based on conventional MCI criteria combined with the SCD questionnaire may be important causes of prevalence variability in CI and CF subtypes in different population studies. The ADAS-Cog scores, typically used as an outcome measure in AD clinical trials (Wattmo and Wallin, 2017), is a better tool for differentiating pre-MCI SCD from MCI and RCF from PRCF. Furthermore, demographic factors had a minor influence on ADAS-Cog scores. A previous study demonstrated the adequacy of the ADAS-Cog scores for assessing psychometric properties in older low-literacy adults in sub-Saharan Africa (Paddick et al., 2017). However, a small-sample study showed that the ADAS-Cog scores were significantly affected by age and education (Ben Jemaa et al., 2017). The different effects of demographic factors on the ADAS-Cog scores, as demonstrated in our study, require further investigation in the future.

Objective criteria for pre-MCI SCD used in the present study have previously been verified to provide more sensitive criteria for individuals at risk for progression to MCI, indicate early pathological alterations in the brain (Thomas et al., 2020), and improve diagnostic precision, biomarker associations, and progression rates of MCI (Bondi et al., 2014; Edmonds et al., 2015a). While determining our *z*-scores criteria, we only replaced the Auditory Verbal Learning Test with a relatively brief measure—the HVLT-R (Ruan et al., 2020b). Moreover, the normative *z*-scores of the remaining three neuropsychological test batteries (digit span forward or backward and digit symbol) further verified the abovementioned objective cognitive criteria. Therefore, we conclude that objective cognitive criteria were better than clinical criteria for classifying MCI and pre-MCI SCD as well as RCF and PRCF. The diagnostic errors caused by clinical criteria for CI and normal cognition decreased after covariate adjustment.

Few studies have operationally defined pre-MCI SCD (Jessen et al., 2014; Solfrizzi et al., 2017; Margioti et al., 2020; Ruan et al., 2020a), and SCD is often substituted for pre-MCI SCD (Rami et al., 2014; Rabin et al., 2015), particularly for preclinical Alzheimer’s disease (Ávila-Villanueva and Fernández-Blázquez, 2017; Lin et al., 2019). The composite score can be disproportionally influenced by poor cognitive performance on only one test; our pre-MCI SCD criteria required two impaired scores, including process scores (1 SD below or above the normative mean) in two different cognitive domains, thereby significantly improving the sensitivity and specificity of the pre-MCI SCD diagnosis (Thomas et al., 2018). Meanwhile, the clinical MCI criteria also included SCD, which further increased the diagnostic errors of pre-MCI SCD and MCI. SCD occurs with different objective cognitive function trajectories, which could result from various causes (Ávila-Villanueva and Fernández-Blázquez, 2017; Jessen et al., 2020). Reversible SCD is related to depressive symptoms, personality features, side effects from medication, and intermittent sleep disturbances (Reid and Maclullich, 2006; Jessen et al., 2020). Furthermore, our results showed that the RCF group experienced significantly higher depression scores than the MCI group (Table 6). Thus, the SCD-Q MyCog score may be a primary source of diagnostic errors. Conventional MCI criteria may be another source of diagnostic errors. The global CDR is not sensitive to MCI severity and prognosis (Chang et al., 2011), is susceptible to recall bias, and is influenced by psychiatric factors (Saxton et al., 2009). Furthermore, the MMSE and SCD-Q MyCog scores significantly improved in categorizing individuals with CI from those without CI after covariate adjustment (Tables 2, 4). However, the adjustments did not indicate an improvement in discriminating the cognitive status among RCF, PRCF, pre-MCI only, and MCI only groups (Tables 5, 6). Instead, the ADAS-Cog scores indicated better consistency with the neuropsychological test scores and could discriminate *z*-score-derived MCI and pre-MCI SCD from cognitively normal and robust normal controls (Table 2), CF from only PF/PPF and normal groups (Table 4), and RCF from other subgroups (Table 6). These findings indicate that clinical criteria results in several diagnostic errors in CF subtypes. In addition, the scores of non-cognitive measures were significantly higher in individuals of the PRCF group than in those of the RCF group and in individuals of the CF group than in those of the PF/PPF, only MCI, only pre-MCI SCD, and normal groups.

A limitation of this study was the small sample size of normative *z*-scores from robust normal controls (Ruan et al., 2020b), resulting in a failure to discriminate pre-MCI SCD from cognitively normal individuals in *z*-scores of intrusion errors and retroactive interference. Increasing the sample size of normative *z*-scores from robust normal controls from community-dwelling individuals will improve diagnostic accuracy. Another limitation was the small sample size of the MCI only and pre-MCI SCD only groups. In addition, although the normative *z*-scores contain the memory, language, and attention/executive domains, the visuospatial domain was not evaluated in our sample, and this limits the possibility to detect the deficits of the visuospatial domain. The visuospatial domain should be evaluated in subsequent studies. Nevertheless, some tests, such as digit span forward, digit span backward, digit symbol, and TMT A for attention or processing speed, also indicated consistent changes.

In summary, the use of clinical criteria for distinguishing MCI from pre-MCI SCD and cognitively normal individuals resulted in numerous diagnostic errors. Covariate adjustment could improve the discriminating ability of clinical cognitive measures. The combination of clinical criteria with objective criteria is implementable and cost effective and will considerably reduce the number of diagnostic errors in CI and CF subtypes in clinical practice.

## Data Availability Statement

The original contributions generated for this study are included in the article/Supplementary Material, further inquiries can be directed to the corresponding authors.

## Ethics Statement

The studies involving human participants were reviewed and approved by the study protocol was approved by Hudong Hospital Research Ethics Committee, Fudan University. The patients/participants provided their written informed consent to participate in this study.

## Author Contributions

QR and ZY: conceptualization. QR, ZY, and JC: data curation. QR, WZ, and JR: analysing and interpretation of data. QR, WZ, JR, JC, and ZY: investigation and methodology. QR: original writing. ZY and JC: reviewing and editing. All authors contributed to the article and approved the submitted version.

## Conflict of Interest

The authors declare that the research was conducted in the absence of any commercial or financial relationships that could be construed as a potential conflict of interest.
