# Supplementary material for: Clinical and Objective Cognitive Measures for the Diagnosis of Cognitive Frailty Subtypes: A Comparative Study
Source: Front Psychol. 2021 May 24;12:603974. doi: 10.3389/fpsyg.2021.603974 (PMC8182758; doi:10.3389/fpsyg.2021.603974)
Supplement: Supplementary file 1 [file Table_1.DOCX]

Supplementary Table 1. The robust and experimental samples on z-scores of individual neuropsychological tests (mean ± SD)

|  | Z-scores derived MCI (n=64) | Z-scores derived  (n= 30+28=61) | Z-scores derived normal  (n=66) | Robust normal ^16^ |
| --- | --- | --- | --- | --- |
| Trail Making：A | 2.645 (5.193) | 1.046 (2.940) | -0.103 (1.774) | -0.0569 (0.794) |
| Trail Making：B | 0.748 (1.463) | -0.0895 (0.488) | -0.110 (0.465) | -0.050 (0.858) |
| HVLT-R: Delayed free recall | -0.863 (0.813) | -0.675 (0.793) | -0.0594 (0.726) | 0.0209 (0.974) |
| HVLT-R: Recognition | -0.844 (1.378) | -0.376 (0.945) | -0.141 (1.159) | 0.0262 (0.960) |
| HVLT-R: Learning slope | -0.329 (0.949) | -0.503 (1.018) | 0.139 (0.877) | 0.0317 (0.941) |
| HVLT-R: Intrusion errors | -0.128 (1.050) | 0.0407 (1.043) | -0.221 (0.864) | -0.0309 (0.946) |
| HVLT-R: Retroactive interference | -0.183 (1.076) | -0.199 (1.219) | -0.157 (0.538) | 0.0238 (0.964) |
| Boston Naming Test Total Scores | -1.247 (1.544) | -0.516 (1.131) | -0.211 (0.982) | 0.0538 (0.797) |
| Animal Fluency total scores | -0.975 (0.752) | -0.536 (0.737) | -0.128 (0.733) | 0.0204 (0.975) |
| Digit Span-Forward Total Trials | -0.734 (1.248) | -0.367 (0.997) | -0.231 (1.284) | 0.036 (0.925) |
| Digit Span-Backward Total Trials | -0.514 (1.123) | -0.226 (0.954) | -0.032 (1.260) | 0.0179 (0.980) |
| WAIS Digit Symbol Total Items | -0.806 (1.226) | -0.301 (0.789) | -0.082 (1.152) | 0.0180 (0.980) |

n= Number of subjects with data; SD= standard deviation
